# Identifying research gaps and priorities for African family medicine and primary health care

**DOI:** 10.4102/phcfm.v16i1.4534

**Published:** 2024-05-06

**Authors:** Klaus B. von Pressentin, Robert Mash, Sunanda C. Ray, Jean-Pierre Fina Lubaki, Innocent K. Besigye

**Affiliations:** 1 Department of Family, Community and Emergency Care, Faculty of Health Sciences, University of Cape Town, Cape Town, South Africa; 2 Division of Family Medicine and Primary Care, Department of Family and Emergency Medicine, Faculty of Medicine and Health Sciences, Stellenbosch University, Cape Town, South Africa; 3 Department of Medical Education, University of Botswana, Gabarone, Botswana; 4 Department of Family Medicine and Primary Health Care, Protestant University of Congo, Kinshasa, Democratic Republic of the Congo; 5 Department of Family Medicine, Makerere University, Kampala, Uganda

## Introduction

The Primary Care and Family Medicine Network (PRIMAFAMED) represents a well-established regional network of academic family medicine departments in the sub-Saharan African region.^1^ Country representatives who participated in the 2023 PRIMAFAMED meeting in Johannesburg, South Africa, on 15 and 16 August 2023 revisited the recommendations from a 2014 network meeting, which described research priorities based on what was known almost a decade ago.^2^ Nineteen people from 10 African countries and two European countries participated in the workshop (Table 1). The authors of this report are journal editors from two African primary health care (PHC) journals, the *African Journal of PHC and Family Medicine* (PHCFM) and the *South African Family Practice Journal* (SAFP), who facilitated the workshop during the 2023 meeting.

**TABLE 1:** Workshop participants.

Country	Number
Belgium	1
Botswana	1
Democratic Republic of Congo	1
Eswatini	1
Ethiopia	1
Ireland	1
Kenya	1
Lesotho	1
South Africa	6
Tanzania	1
Uganda	1
Zambia	3

**Total**	**19**

## Workshop process

A three-step process (Table 2) led to this report on the final consensus.

**TABLE 2:** Workshop process overview.

Step	Main activities	Components
Step 1: Three contributions	Workshop participants considered three contributions presented during the PRIMAFAMED meeting.	The editors-in-chief presented an analysis of African family practice and primary care research published in the PHCFM and SAFP journals.^3,4^ An opening address by a senior World Health Organization (WHO) Afro policy maker, Dr Humphrey Karamagi, and a reflection on the changing disease burden in this region.^5^ A summary of the 2014 network meeting’s findings and recommendations on research priorities.^2^
Step 2: Group discussion in two parts (each part consisting of a 30-min discussion and 15-min reporting back)	Discussion on research priorities. Recommendations for capacity-building activities.	A discussion of research priorities based on the three contributions listed above and each person’s knowledge of their country’s context. Recommendations for capacity-building strategies that could be implemented through PRIMAFAMED.
Step 3: Analysis	Collation of key points from group discussions. Analysis using a combined matrix.	The facilitators captured and collated the key points from the group discussions on newsprint and made their field notes. The authors used a combined matrix to analyse the feedback that integrated the WHO PHC measurement framework and the primary care research typology described by Beasley and Starfield (see Table 3).^6,7^

PRIMAFAMED, Primary Care and Family Medicine Network; SAFR, South African Family Practice Journal; PHCFM, South African Journal of Primary Health Care & Family Medicine.

Note: Please see the full reference list of the article, Von Pressentin KB, Mash R, Ray SC, Fina Lubaki J-P, Besigye IK. Identifying research gaps and priorities for African family medicine and primary health care. Afr J Prm Health Care Fam Med. 2024;16(1), a4534. https://doi.org/10.4102/phcfm.v16i1.4534, for more information.

## Overview of the contributions during Step 1

### The analysis of African family practice research

Two recent reports described an analysis of research published during 2020–2022 in the PHCFM and SAFP journals.^3,4^ Although publications had a median number of three authors, most research was derived from only one institution or discipline, indicating a need for collaborative and interdisciplinary research. Most authors were from South Africa (80%), implying a relative lack of published research from other countries in sub-Saharan Africa. The research mainly focused on health services with little on broader PHC issues such as community engagement or multisectoral action. Clinical research focused on infectious diseases, non-communicable diseases, maternal and women’s health, with little focus on mental health care, injury and trauma, palliative care and rehabilitation. Service delivery research addressed person-centredness and comprehensiveness of care, and noticeable gaps included research on continuity, care coordination, effectiveness and efficiency. There was a neglect of research on children, and almost all studies were descriptive, with little publication of observational or experimental work.

### The World Health Organization’s Afro Regional perspective

Dr Karamagi highlighted several implications for education and research on PHC needs for African health systems.^5^ He emphasised the link between a PHC approach, universal health coverage (UHC) and meeting the sustainable development goal of ‘Good health and well-being for all ages’. He argued that this requires ‘tangible hardware’ (health workforce, infrastructure and products), ‘tangible software’ (delivery and information systems, finance and governance processes) and, importantly, ‘intangible software’ (relationships, networks, values and norms to inform beliefs and practices). In terms of research priorities, he highlighted the need to develop and evaluate different models of care to strengthen service delivery, to consider what kind of health workforce is needed and the contribution of family physicians to strengthening healthcare teams.

### Research priorities from the 6th Primary Care and Family Medicine Network meeting

The authors of the 2014 report^2^ highlighted strengths such as growing support and leadership to drive research activities and an established culture of networking and collaboration. Weaknesses that hindered the growth of primary care research included limited capability and capacity, failure to publish and disseminate findings, poor coordination, lack of innovation in research projects and study designs and lack of support from policymakers in academic and government spheres. The report highlighted strategies to build capacity for primary care research from three perspectives: regional and international networks, individual countries and educational institutions and family medicine departments.

## Research priorities generated during Step 2

The combined matrix in Table 3, which amalgamated the WHO PHC measurement framework and primary care research domains, was used to integrate the group work feedback.^6,7^

**TABLE 3:** Matrix that integrates the typology of primary care research and the WHO measurement framework for PHC.

Typology of primary care research^7^	WHO PHC monitoring conceptual framework^6^			
	*PHC components* (integrated health services, multisectoral policy and action, empowered people and communities)	*Health system determinants* (structures and inputs)	*Service delivery* (processes and outputs)	*Health system objectives* (outcomes and impact)
Basic research	Developing the tools or methods to conduct research, such as coding systems and data-gathering instruments.	Developing the tools or methods to conduct research, such as coding systems and data-gathering instruments.	Developing the tools or methods to conduct research, such as coding systems and data-gathering instruments.	Developing the tools or methods to conduct research, such as coding systems and data-gathering instruments.
Clinical research	Diagnosis and management of common primary care conditions, including multimorbidity.	Overarching healthcare structures and policies impacting clinical care.	**Quality of care:** Technical quality of care for different conditions, effectiveness of clinical care and interventions for specific conditions.	Clinical outcomes and impact.
Health services research	Integrated services Community engagement Multisectoral action	Overarching healthcare structures and policies impacting health services delivery.	**Processes:** models of care, systems for improving quality, resilient health facilities and services.	Health services outcomes and impact.
			**Outputs:** access and availability, quality care, including core primary care functions, patient safety and efficiency of care.	
Health systems research	Multisectoral policy	**Structures:** Governance, adjustment to population needs, financing	The interactions between primary care service delivery and PHC-orientated health systems.	**Evaluation of health system objectives:** coverage, financial protection, security, health status, responsiveness, equity.
		**Inputs:** infrastructure, health workforce, medicines and other health products, health information, and digital technologies.		
Educational research	Overarching research evaluating the interplay between health and educational systems within different community contexts.	**Health workforce:** appropriate healthcare workforce training, including promoting primary care careers.	**Quality of care:** competence of healthcare workers, including continuing professional development.	Impact of educational system and outputs on health outcomes.

PHC, primary health care.

Note: Please see the full reference list of the article, Von Pressentin KB, Mash R, Ray SC, Fina Lubaki J-P, Besigye IK. Identifying research gaps and priorities for African family medicine and primary health care. Afr J Prm Health Care Fam Med. 2024;16(1), a4534. https://doi.org/10.4102/phcfm.v16i1.4534, for more information.

### Basic research

The groups did not specify typical basic research issues, such as the need to develop research tools or instruments. However, they highlighted the need to diversify our methodological approaches, including building bridges with complementary research fields, such as public health, and ensuring that interdisciplinary collaborations investigate cross-cutting focus areas. This will enable research capacity-building and the development of research methods and paradigms, such as implementation science and action research.

### Clinical research

Although all aspects of the burden of disease are relevant, there may be a need to consider multimorbidity, mental health, violence and trauma as relatively neglected areas. In addition, more attention should be given to the extremes of age in terms of children and older adults. Researchers should continue their work on disease prevention and behaviour change, and focus more on palliative care and rehabilitation. These new priority areas also offer opportunities for interdisciplinary research teams.

### Health services research

#### Service delivery processes

There is a desire to describe different PHC models across various contexts, mainly focusing on defining the package of services and the role of family physicians and other providers in these models of care. There was a specific emphasis on raising the profile of family physicians to allow policymakers to grasp the additional value they bring compared to non-specialist primary care clinicians in strengthening teams and services. Focus areas also include community engagement and participation, understanding palliative care and rehabilitation in these models of care and leadership capabilities needed in service delivery. The groups also considered broader societal and environmental forces, such as planetary health, which may impact the quality and resilience of PHC services and facilities.

#### Service delivery outputs

The groups agreed that research must centre on the core primary care functions, such as access, coordination, continuity, comprehensiveness and person centredness.

### Health systems research

#### PHC components

We must expand our focus to broader PHC research, including community empowerment and multi-sectoral action as key PHC components. These components are integrated health services, focusing on combining primary care and public health functions to deliver comprehensive care, the significance of multisectoral policies and actions to address broader determinants of health, such as social, economic, and environmental factors, and the empowerment of individuals and communities to advocate for health-promoting policies and actively collaborate in the development of health and social services.

#### Health system structures

Research on the financing of primary care could build on Starfield’s previous work,^8^ especially from the African region in the post-COVID-19 era.^9^ More updated evidence is needed on the funding of PHC.

#### Health system inputs

The group work especially highlighted the following facets of health system inputs: health workforce (team composition, tracking graduates and other human resources for health issues), health information (using local and global data to inform local priorities and planning, especially to assess community health needs and to implement community orientated primary care) and digital technologies for health (including mobile apps and drones to enhance rural care and agricultural practices).

#### Health system objectives (outcomes and impact)

Participants confirmed the need to continue evaluating the role of family physicians in improving health system performance and health outcomes.

### Educational research

The call to transform health professions education to better cater to population health needs necessitates a departure from conventional undergraduate and postgraduate teaching. This transformation entails a more comprehensive approach to integrating community orientation into the curriculum through experiences embedded in PHC services. Rather than solely focusing on individual patient care, this new paradigm emphasises the broader societal and environmental factors impacting health outcomes. By embracing transformative education, health professionals can better understand population health dynamics and collaborate more effectively with PHC stakeholders to address these complex issues.^10^ A differentiated approach to models of care should span both public and private healthcare sectors, which warrants work around understanding family physician roles, training and career paths in these different models and sectors.

## Capacity-building priorities generated during Step 2

The group work findings were organised according to capacity-building topics and interventions, and stakeholders or actors who should help implement the proposed interventions.

### Capacity-building topics

Foundational topics include building capability across more diverse study designs and methods and data analysis using software packages. Competencies in academic writing and peer-reviewing skills were suggested, as well as grant writing and science communication to influence policy and advocacy. There is a need to develop capacity around using data in clinical governance, such as in clinical audits and quality improvement. Skills in coordinating larger multicentre teams and projects will attract more substantive funding and produce higher-quality evidence. Such teams may also include methods experts with specific expertise in study designs to answer complex questions. There is a particular need to build postgraduate research supervision and examination capacity, especially around doctoral research.

### Capacity-building strategies

Interventions at the collective level include educational interventions, such as online and conference workshops, and the sharing of resources. There is a need to foster collaborative research projects, including practice-based research networks and multi-country projects. The concept of research lab models such as those encountered in laboratory sciences was suggested. In these models, the principal investigator grows a laboratory team centred around a nucleus of established and senior researchers supervising a mixed group of early career researchers ranging from master’s to postdoctoral levels. These models help build a network of researchers at different career stages, facilitating peer and near-peer learning and mentoring. These models may be adapted to PHC research teams, allowing multidisciplinary teams at institutional and national levels to develop around specific areas of interest. Such teams may also serve as incubators for research by bringing together researchers and clinician-scholars with different areas of expertise, including implementation science methodologies.

Postgraduate research interventions linked to academic institutions include collaborations around building supervision capacity, like initiatives by the South African Academy of Family Physicians’ (SAAFP) PhD special interest group, the Consortium for Advanced Research Training in Africa (CARTA) and other global South-South collaborations.^11,12^ Other interventions include co-badged degrees, cohort PhD programmes, and shared supervision models. Growing a scholarly nucleus of emerging and established researchers at departmental, national and regional levels will help to strengthen the discipline and increase its profile. There is also a need to develop research supervision expertise to support dissertation and publication outputs. This range of research and supervision-related collaboration models will attract more substantive funding to support capacity building, more complex research designs and dedicated time for clinician-scholars to focus on their research.

### Stakeholders with the potential to implement these interventions

Primary Care and Family Medicine Network is a central stakeholder to guide the implementation of these proposed interventions. It should expand its current offering of workshops to include online platforms to share resources and increase the reach of its listserv. The participants suggested that the network should evaluate the impact of capacity-building activities in supporting researchers at different career stages. Such evaluations should seek the views from previously underrepresented countries.

At departmental and national levels, family medicine departments should cultivate research teams consisting of clinician-scholars and researchers from different disciplines and career stages. Departments should also map supervision capacity within and across universities to identify centres of expertise and those needing support. Such a mapping exercise will develop a supervisor database. Academic departments should explore the options available for clinicians and jointly appointed academics to ensure access to dedicated research funding and time to grow as clinician-scholars. Lastly, departments should also examine the possibilities in their institutions to fund article processing charges (APCs) and data analysis software access.

The workshop participants listed suggestions for scholarly journals in family medicine and PHC, including supporting authors from PRIMAFAMED with reduced APCs. They also suggested a new special collection on research methods and supervision competencies to build on the previously published PHCFM series.^14^ The journals could also commission reviews on under-researched areas and commentaries that summarise published research findings in the African region.

## Ten years later – Step 3: An updated reflection on research priorities and capacity-building needs

Much has changed since the 2014 report, including the 2018 Astana PHC recommitment, the 2022 WHO PHC measurement framework release, and a global pandemic that has challenged the foundations of all nations and their health and economic sectors.^6,9,15^ When comparing the two snapshots of 2014 and 2023 from the PRIMAFAMED perspective, it is worth reflecting on our research priorities and whether we need to adjust our strategies to meet the changing needs of our network and region. Table 4 describes core priorities and strategies to inform the network’s agenda over the next decade. This report is limited as it captures the output of a single workshop whose participants only represented part of the sub-Saharan African region. Other methods, such as the recently used Delphi design, may be considered for future consensus-building activities.^15^ However, the workshop and its report represent the work of key PRIMAFAMED role players with the agency to implement the identified strategies.

**TABLE 4:** The Primary Care and Family Medicine Network research and capacity-building priorities for the next decade.

Research priorities	Capacity-building needs and strategies
The core dimensions of primary care are now more centre stage, which may be attributed to the increased use of the Primary Care Assessment Tool in the region.^16^ Showcasing the value of family physicians in strengthening team-based primary care services remains a priority for educational, health services and health systems research lenses. The African Surgical Outcomes Study (ASOS) was suggested as an example of multi-country research highlighting unmet surgical needs and managing postoperative complications as under-researched areas in primary care.^17^ More work is needed to highlight the role of family physicians in expanding access to district-level surgical services.^18^ There is an increased awareness of the need to perform research which bridges primary care and PHC whole-society issues in the wake of the COVID-19 pandemic and the increasing effects of planetary health issues on upstream factors such as food security.^19^ This integrated primary care and public health research approach should also focus on health system structures and inputs, such as financing and information systems, as well as the emerging priority of digital technologies for health. The participants did not flag other health systems inputs, such as physical infrastructure, medicines and health products. This highlights the value of using all the components of the PHC framework to inform a research agenda that will address some of our discipline’s possible blind spots.	As mentioned in the previous report, the development of capability and capacity depends not only on training but also on experience and maturation throughout a career. The training needs identified during the 2023 meeting revolve around advanced research training, such as relevant methodologies, including implementation science, and academic writing for different audiences, such as scientific journals, policymakers and funders. Partnerships with health services, communities, and policymakers will ensure social accountability and relevant research activities. Establishing and growing a research culture at departmental and broader organisational levels will be possible through larger multi-country projects and practice-based research networks. These network activities continue to fall within the purview of national, regional, and international family medicine and primary care bodies, such as PRIMAFAMED and WONCA, which see increasing south-south collaboration. These networks could also offer mentoring opportunities for early career researchers across countries with different capacity levels, such as an online programme designed to improve research activity among early-career family physicians in the African region.^20^ A particular need remains to strengthen postgraduate researcher development, including doctoral research and supervision activities. The goal of at least one person with a PhD per department who can supervise and capacitate other researchers remains aspirational. The SAAFP PhD special interest group has started mapping doctoral supervision capacity and needs nationally.^11^ This mapping approach could be expanded across PRIMAFAMED, representing an opportunity to perform a baseline measurement and track progress over the next decade. The previous report highlighted the usefulness of the 10 primary care research methods articles published in the PHCFM during the same year.^2,13^ The 2023 workshop participants suggested a follow-up special collection on research methods and competencies for doctoral researchers and supervisors.

PRIMAFAMED, Primary Care and Family Medicine Network.

Note: Please see the full reference list of the article, Von Pressentin KB, Mash R, Ray SC, Fina Lubaki J-P, Besigye IK. Identifying research gaps and priorities for African family medicine and primary health care. Afr J Prm Health Care Fam Med. 2024;16(1), a4534. https://doi.org/10.4102/phcfm.v16i1.4534, for more information.

## Conclusion

This workshop report provides an updated PRIMAFAMED assessment of current research and capacity-building priorities for family medicine and primary care in African PHC-orientated health systems. Research priorities have expanded to a comprehensive PHC perspective. Despite some progress, there remain opportunities for the network, its affiliated journals, and other partners and stakeholders to strengthen primary care research capability and capacity in the African region.
